# Clinical Epigenetics on the Baltic Coast

**DOI:** 10.3390/epigenomes6030019

**Published:** 2022-07-20

**Authors:** Ryszard Maleszka

**Affiliations:** Research School of Biology, The Australian National University, Canberra, ACT 2601, Australia; ryszard.maleszka@anu.edu.au

**Keywords:** DNA methylation, epigenetic biomarkers, cancer epigenomes, ageing, epigenetic disease

## Abstract

This report summarizes the proceedings of the inaugural Clinical Epigenetics Conference that was held in Szczecin, Poland, from 8 June 2022. With focus on epigenetic diseases whose causes, progression, and prognosis are associated with aberrant epigenomic alterations, the meeting was a timely forum to discuss recent progress in this rapidly evolving field and consider avenues for converting experimental data into clinical reality that would be beneficial for patients. The wealth of the presented data was an impressive showcase of the enormous challenges faced by researchers in their quest for understanding the benefits and limitations of the available information in the medical context. A shared view among the participants was that merging the current state of knowledge with clinical applications will be promptly achieved.

## 1. Introduction

What are the promising future advances in clinical epigenetic research? How can epigenetic research complement other approaches in clinical medicine, and what surprises does it hold for disease treatment? Will epigenomic biomarkers make an impact on disease diagnosis and prevention? What are the prospects of developing personalized epigenetic medicine? These were just some of the topics discussed at the inaugural Clinical Epigenetics (CLEPIC) conference held in the major Baltic seaport of Szczecin in Poland [1].

Organized by the Clinical Epigenetics and Epigenetics Communications journals, CLEPIC covered many aspects of a broadly defined epigenetics and included not only clinically relevant presentations, but also other topics concerned with basic epigenetic mechanisms and evolution. In addition to science, the spirit of solidarity with the people of Ukraine facing the Russian invasion was another unifying theme of this event. A historical venue of the Pomeranian Medical University combined with the hosts’ hospitality and an enjoyable social program provided ample opportunities for productive interactions. At the close of the conference, the organizers received many warm compliments on the success of the well planned, smoothly run, professional, and indeed very enjoyable event. As with ribozymes carrying out phosphoryl transfer reactions, comfortable and well-catered social environments serve to catalyze a healthy exchange of scientific ideas. This report summarizes, from the abundance of information presented at this meeting, the major lines of research with the clinical potential along which the emerging field of clinical epigenetics is progressing. The talks were arranged in seven sessions: Epigenetic Mechanisms, Epigenetic disease, Epigenetic biomarkers, Cancer Epigenetics, Epigenomic Approaches Across the Tree of Life, Computational Analysis of Epigenomes, and Development and Ageing.

## 2. Conference Topics

One aim of the conference was to facilitate the development of a more cohesive international community that would focus on the study of epigenetic mechanisms in disease and forthcoming applications. This sentiment was part of welcoming remarks by two editors of the organizing journals, Marianne Rots (Groningen) and Lucia Altucci (Naples), who also saw the potential of this inaugural gathering to become an annual forum to foster translational clinical epigenetics. The opening keynote talk was given by Susan Clark from Sydney, who presented a comprehensive overview of her DNA methylation studies in the context of disease (especially in cancer). Indeed, her talk set the scene for many presentations focusing on epigenetic investigations in cancers (Carmen Jerónimo, Porto; Monika Hegi, Lausanne; Pamela Munster, San Francisco), type-2 diabetes (Charlotte Ling, Malmö; Harol Snieder, Groningen), melanoma (Paola Arimondo, Paris), behavioral effects (Moshe Szyf, Montreal), SARS-CoV-2 infection (Wojdacz, Szczecin), and chronic obstructive pulmonary disease (Renata Jurkowska, Cardiff). Remarkably, virtually all analyzed methylomes and other examined layers of epigenomic regulation show well-defined differences between the disease and control situations, suggesting significant alterations at the epigenomic level with often easily detectable changes in gene expression. While the mechanistic aspects of these epigenomic modifications are not fully understood, the results are promising and merit future studies to facilitate potential translational approaches.

In contrast to most human tissues that are diploid, tumors such as melanoma are exceedingly heterogeneous at the cellular level, which results in each tumor being unique in terms of its combination of variants and structural alterations. To overcome this challenge, Arimondo hypothesized that melanoma aggressiveness is associated with a common signature of hypermethylation that appears early in tumorigenesis and is shared in aggressive cells. This line of reasoning allowed her lab to identify eight hypermethylated promoters for validation in melanoma patients. Given a significant level of cellular complexity of mammalian tissues and the multiple epigenomes underlying such diversity, several presenters have taken great care to generate high resolution data in single cells (sc). Henk Stunnenberg (Utrecht) used sc-RNA and sc-ATAC sequencing to unravel gene expression and enhancer activities in LPS-challenged human bone marrow that is used as a model for sepsis. Changes in the expression of interferon type-I in myeloid and lymphoid lineages were uncovered along with a loss of intermediate monocytes underscoring the importance of type-I interferon in pathology of sepsis.

Several computational and statistical approaches to the challenging issue of cell-type heterogeneity and other aspects of large-scale epigenomic studies were addressed by experts in the session devoted to in silico analyses (Simon Heath, Barcelona; Joost Martens, Nijmegen: Andrew Teschendorff, Shanghai). Given the exponential accumulation of raw epigenomic data in GenBank, several examples of successful protocols developed by these labs to deconvolute the complexities of epigenomic regulations are reassuring.

Epigenetic regulation is driven by many interlocked processes with dozens of modifying proteins operating at the DNA and chromatin levels. While some progress has been made in unravelling the biochemical properties of these epi-toolkits, we are still largely ignorant of many details of their modi operandi. This important issue was the topic of Albert Jeltsch’s (Stuttgart) talk in which he showed how a “deep enzymology” approach helps to unravel the impact of flanking sequences on enzymatic activities of both human and mouse DNA methyltransferases DNMT3A and DNMT3B, as well as TETs (dioxygenases involved in 5 mC to 5 hmC conversion). Strong impacts on the type of CpG or non-CpG methylation activity, structure of DNMT-DNA complexes, and DNMT3A/DNMT3B preferences suggest that specific DNA methylation patterns are partly determined by flanking sequences.

Examples of diverse evolutionary inventions in epigenetics and their impacts on genome and transcriptome functions were described in invertebrates (Ryszard Maleszka, Canberra), and plants (Frank Johannes, Munich; Isabel Bäurle, Potsdam). These lineage specific epi-toolkits offer important insights into epigenetic mechanisms responding to environmental stressors. Additionally, invertebrates such as nematodes are associated with many human diseases and their unique epi-toolkits could be used as targets for drug development.

Many health disorders previously referred to as “complex” are now considered to be of epigenetic origin. Cancer, mental disorders, neurodevelopmental syndromes, certain types of metabolic diseases, and many others cannot be easily explained as simple Mendelian traits. They often develop over lifetimes, vary in severity, and are shaped by hundreds of gene products under the epigenetic influences of external factors. In this context, an important challenge for clinical epigenetics concerns predictions on the future of healthy individuals who might be affected by these types of illnesses. In a talk about epigenetic biomarker discovery, Maria Berdasco (Barcelona) brought into focus several impediments hindering the progress in converting preclinical findings to clinical applications. Epigenetic diversity in human populations and the multilayer nature of epigenomic machinery are the key obstacles for selecting optimal targets and designing universal biomarkers. In a subsequent presentation, Bożena Kamińska (Warsaw) described an integrative analysis targeting 100 epigenetically regulated genes to create an atlas of active enhancers and promoters in benign and malignant gliomas. She emphasized the importance of non-invasive liquid biopsies in analyzing tumors to improve early detection and patient survival.

Although disease biomarkers offer a most promising therapeutic option for personalized epigenetic medicine, epi-drugs targeting selected gene products or epigenetic mechanisms and their responsiveness to modifiers can also be exploited as therapeutic agents. However, a search for novel epigenetically active compounds could be a long and tormenting process as described by Angel de Lera (Vigo, Spain) in his talk accounting the process of synthesizing nahuoic acid, an inhibitor of a regulator of cell cycle progression SETD8 (histone lysine mono-methyltransferase).

One area in which the predictive power of genome-wide epigenomic profiles is yielding promising results is ageing. Both chronological and biological ageing are associated with epigenetic clocks that can be visualized with DNA methylation profiles. This session was opened by Alex Meissner (Berlin) with a comprehensive overview of his group work on epigenomic mechanisms in genome regulation with the main focus on DNA methylation dynamics in early development, and cell reprogramming. Riccardo Marioni (Edinburgh) presented a rather impressive set of data based on a large cohort of participants and described his methodology to refine the prediction accuracy (median error 2.3 years). Wolfgang Wagner (Aachen) reinforced this concept by showing his lab’s efforts to make the epigenetic clocks even more robust. One interesting finding from these studies is that certain genomic regions that maintain age-associated DNA methylation across both strands are similar in humans and mice suggesting a conservation of the underlying mechanism between lineages. Although a mouse is not a human being, these types of conserved mechanisms reassert the practicality of the mouse model to study the effects of epigenomic disorders in humans.

The last keynote lecture by Wolf Reik (Cambridge) was an impressive tour de force of multi-omics analyses in single cells during early mammalian development, especially during epigenetic reprogramming. He reported on new insights into transcriptional dynamic including priming and acute remodeling of enhancers during the transition from pluripotency to initial cell fate decisions before gastrulation. He also highlighted his group’s interest in the programmed degradation of epigenetic information during ageing and how this process is coordinated across tissues.

Many interesting talks were given by a contingent of younger researchers including several early career investigators whose work was selected for oral presentations from submitted abstracts. Those who missed this opportunity had their projects highlighted in a vibrant poster session.

## 3. Concluding Remarks

At the end of the conference, I was left with a picture of a rapidly evolving research field producing massive, often overwhelming, amounts of epigenomic data, which are in dire need to be converted into clinical applications. As pointed out by Stephen Beck (London) in the second keynote lecture, many hurdles hamper the progress of translating epigenomic findings into medical and commercial reality.

With many existing diseases and new threats on the rise, the quest for novel diagnostic tools and efficient treatments will only become more pressing over time and will be a primary challenge for the translational epigenetic research. Although the datasets presented during the conference provide a compelling platform for testing their clinical utilities, the key challenge is to determine which of those epigenomic, or transcriptomic perturbations are causal, and which are irrelevant to anomalies brought about by disease states. A critical mass of clinicians, biologists, bioinformaticians, biochemists, and software companies crafting and integrating new analytic tools is required to reach the desired momentum. A general feeling of confidence was shared among the participants that this goal can be promptly achieved, and new therapies will be developed for patients.

In the long-term, the acid test will be the usefulness, or the lack thereof, of the lab results.
